# Uterine Fibroid Embolization in a Series of Women Older Than 50 Years: An Observational Study

**DOI:** 10.1089/whr.2021.0057

**Published:** 2022-02-28

**Authors:** Benedetto Mondelli, Woodruff John Walker, Tanveer Dhanoya, Karen Morton

**Affiliations:** ^1^ Department of Obstetrics and Gynaecology, Surrey and Sussex Healthcare NHS Trust, Redhill, United Kingdom.; ^2^ Interventional Radiology, Royal Surrey County Hospital, Guildford, United Kingdom.; ^3^ Department of Obstetrics and Gynaecology, Royal Surrey County Hospital, Guildford, United Kingdom.

**Keywords:** uterine artery embolization, postmenopausal, uterine fibroids

## Abstract

***Objective:*** Women older than 50 years, and in particular postmenopausal, are not usually considered candidate for uterine artery embolization (UAE). We reviewed the outcome of UAE in a series of women older than 50 years, who presented with different symptoms of uterus enlargement.

***Population:*** Women referred to the radiologist from gynecologists in the United Kingdom with minimal age over 50 years.

***Methods:*** This is a retrospective observational study. The clinical criteria are women older than 50 years with symptoms related to large fibroids affecting their quality of life, who underwent UAE over a 4-year period at Royal Surrey Foundation Trust Hospital and London Clinic between 2012 and 2016. We retrieved the information from the patients' hospital notes and respective images, including magnetic resonance and ultrasound of the pelvis, and from questionnaires and telephone reviews.

***Main Outcome Measures:*** We measured the reduction of uterine size, complications, and overall satisfaction of patients.

***Results:*** The overall reduction of uterine size 8 weeks following UAE was between 50% and 64% in 12 out of 23 patients. Thirty-five percent of patients (8/23) experienced a reduction of over 65% of overall uterine volume. Only on 2 occasions, the reduction was below 50%. We asked the patients if they would recommend this operation. Twenty out of 23 would recommend it. Improvement of symptoms was measured with a scale between 0 and 5. Twelve out of 23 had total resolution of symptoms—no change in 1 case. One patient underwent a hysterectomy as symptoms persisted.

***Conclusions:*** In our series of women older than 50 years, UAE was an effective alternative to surgery, with reduction in fibroid size and improvement of symptoms.

## Introduction

We present a unique series of patients older than 50 years (65% postmenopausal) having uterine artery embolization (UAE) for fibroids, and to our knowledge, no such group having fibroid embolization has been studied. Uterine fibroids known as leiomyomas are benign tumors that originate in the uterus. Leiomyomas often appear during childbearing years and only rarely develop into a malignancy. Many women who have fibroids are asymptomatic; others have symptoms influenced by the location, size, and number of fibroids. Depending on the presentation, parity, age, and the woman's preferences, there are currently four main surgical treatment options for uterine fibroids: hysterectomy, myomectomy, transcervical resection of intracavity fibroids, and UAE.

Complications of hysterectomy are well known and researched. More importantly, Reed et al. showed that in patients having a myomectomy, of all ages, the cumulative incidence of second surgery was 21.8% (74.8% of which were hysterectomies).^1^ There are also temporizing medical treatments, such as gonadotropin releasing hormone analogues treatment. Since its initial development in the mid-80s,^2^ UAE has been developed as an alternative, less invasive procedure to shrink fibroid size. Shrinking the size of fibroids should relieve pressure symptoms, and urinary incontinence symptoms, and might potentially reduce the chances of abnormal uterine bleeding.

In comparison, UAE complication rates are less than the surgical alternatives: the infection rate is very much lower than for hysterectomy (1/200); mortality from UAE is negligible.^3^ Other benefits of UAE when compared with myomectomy or hysterectomy are that it is a quicker procedure, thus taking less surgical time and less anesthetic time as it can be done under local anesthetic or sedation, therefore reducing the risk of venous thromboembolism and anesthetic risks for the patient.

Complications and side effects of UAE include the following: a transient vaginal discharge reported to affect up to 16% of patients at 12 months^4^ and infarction and expulsion of fibroid material can occur in up to 10%.^4^ In a small percentage of patients, vaginal discharge can become a chronic problem that can be treated with hysteroscopic resection.^5^ The long-term success rates of UAE is over 90%.^6,7^ Fibroid embolization has been used extensively in premenopausal women. There has been a reluctance to embolize fibroids in postmenopausal women mainly because of the fear of sarcomatous change. A fibroid's behavior after menopause is unpredictable, and although a percentage of fibroids may shrink, many will remain static. Others can grow even though they are benign.^8^

The purpose of this review was to evaluate the local outcomes in our unit of UAE for women complaining of symptoms associated with a bulky uterus, who would usually be offered surgical intervention. We measured this outcome by assessing women's symptoms through a questionnaire, and by measuring reduction in uterine size.

## Methods

We present a single-centered study involving 23 patients older than 52 years, where UAE was performed. Patients were assessed by retrospective analysis, including evaluation of patient notes and imaging (including ultrasound [USS] and magnetic resonance imaging [MRI]). Patient's questionnaire was filled in preoperatively and postoperatively. A telephone consultation was then carried out years following the procedure to investigate the overall patients' satisfaction. Data were analyzed with Microsoft Exel.

The clinical criteria are women older than 52 years with symptoms related to large fibroids affecting their quality of life, who underwent UAE over a 4-year period at Royal Surrey Foundation Trust Hospital and the London Clinic between 2012 and 2016.

## Technique

The decision to proceed to UAE was based on symptoms, imaging, and cases amenable to UAE. Women who presented with irregular bleeding were investigated as per local guidelines to rule out endometrial pathology. Review of up-to-date uterine imaging is fundamental before proceeding with UAE. All patients will have a pelvic USS and MRI to assess the leiomyoma size and position and any other uterine or adnexal anomaly or nongynecological pathology. A magnetic resonance angiogram should also be included to map blood supply.

The USS helps to rule out absolute contraindications for UAE, which include viable pregnancy, active infection, and suspected malignancy. It is established that MRI has become the next mandatory step following USS.

Although uterine leiomyosarcoma is extremely rare, comprising 1.3% of all uterine malignancies, an MRI pelvis is performed to investigate and recognize any sign of potential malignancy arising from the uterine mass.^9–13^ The presumed incidence of occult malignancy in a patient undergoing surgery or UAE is ∼1 in 350 to 1000 or less.^14^ It can be challenging to distinguish between a degenerating fibroid and a leiomyosarcoma as there are no obvious morphological differences that can help with the diagnosis. In case there is doubt, a transabdominal image-guided biopsy may be considered.

UAE is traditionally performed with moderate sedation and local anesthesia through the right common femoral artery approach under X-ray vision. Once the catheter reaches the internal iliac arteries, a microcatheter is passed into the mid-section of the uterine arteries bilaterally, where fine particles of polyvinyl alcohol 355 to 500 microns are injected until distal branch stasis is achieved. Prophylactic intravenous antibiotics are administered before the procedure.

Postoperative pain is controlled with PCA pump and intravenous opiates. The estimated length of stay in the hospital is one to two nights. The first follow-up was 8 weeks following UAE where a pelvic USS was performed to confirm the uterine mass infarction. Following this first follow-up, a second MRI was then performed 6–12 months after the procedure to calculate the overall reduction in uterine size.

## Results

Twenty-four women older than 52 years with uterine fibroids, receiving UAE, were identified in the single-centered study. One patient did not want to take part in this review. The median age was 59 years, with the youngest 52 and the oldest 72.

The vast majority of them complained of pressure and discomfort symptoms (20/23). Other common complaints were urinary symptoms (7/23) and dyspareunia (4/23). Fifteen patients (65%) were postmenopausal, while the others were still experiencing bleeding. The bleeding was regular in six of them, irregular in two cases, and continuous for one patient, affecting the quality of life in five patients. Patients were fully informed about the different surgical and medical options available to treat their symptoms. Women were offered UAE to relieve symptoms, which might have been due to having a bulky uterus such as pressure and urinary incontinence, but they were aware that the symptoms might persist if caused by alternative pathology, and sometimes even in the case of reduction of uterine size.

Two out of 23 patients (8.6%) failed to fill in a preoperative questionnaire.

Four patients had undergone uterine surgery to improve their symptoms in the past, including three transcervical resections of fibroid and two UAEs. One patient tried both procedures in the past. Interestingly, all the patients, except for one, had information about the UAE from a gynecologist and only a single patient researched this procedure independently before the specialist's consultation.

Transvaginal USS and an MRI pelvis were performed for all the patients before the procedure. The sizes of fibroids embolized ranged from 4.8 cm (smallest) to 20 cm (largest).

The volume of the uterus' ranged from 450 mL (smallest) to 4004 mL (largest). The majority of embolizations were performed for a uterus between 500 and 1500 mL (16/23–69.5%).

Fifty-two percent of patients (12/23) experienced a 50%–64% reduction in uterine volume when scanned 8 weeks postprocedure. Thirty-five percent of patients (8/23) experienced a reduction of over 65% of overall uterine volume. Only on 2 occasions, the size reduction was below 50%.

At 8 weeks, a patient questionnaire was filled in by all, except 4 of the 23 patients. One hundred percent of patients, who filled in a questionnaire, found the information given before the procedure exhaustive, and 84% (16/19) found the embolization better than expected. The pain was described as better than expected for 10 out of the 19 patients (52%), whereas 7 patients found it worse than expected. All the treated patients were happy with the 2 days of hospital stay, and 13 patients (68%) experienced a bruise at the incision site.

In most cases (13/19–68%), women required regular analgesia for fewer than 5 days, and only in 2 cases, the patients took oral analgesia for more than 10 days. Three patients experienced postembolization syndrome, treated in all cases with paracetamol and ibuprofen. Seventy-nine percent of patients who filled the questionnaire went back to regular activity after less than 3 weeks.

A common side effect of UAE is vaginal discharge. This was experienced by 14 out of 19 patients (73.6%). In 4 patients, the discharge was still ongoing at the 8-week review and was offensive in 2 cases. Passage of fibroid happened in 2 cases (10.5%). Six to 12 months following the procedure, the uterine volume was rechecked with an MRI pelvis.^15^ Thirteen out of 23 patients (56.5%) had a reduction between 50% and 64%. Thirty-five percent of patients (8/23) experienced a reduction of over 65% of overall uterine volume. Only in 1 case, the reduction was below 50%.

Following this procedure, we contacted the patients, and we asked them if they would recommend this operation if looking back. Twenty out of 23 (87%) would recommend it. Symptom improvement was measured with a scale between 0 and 5, with 0 indicating no change and 5 indicating complete resolution of symptoms. Twelve out of 23 (52%) had a total resolution of symptoms, and there was no change in 1 case (0/5). Score one to four indicating improvement of symptoms in 10 patients (not complete resolution). One patient underwent a hysterectomy as symptoms persisted.

## Discussion

### Main findings

We have shown that embolization in this group of patients is very effective in terms of shrinkage and symptomatic relief.

Patients' satisfaction is an important factor to consider, and only in 1 case, the patient would not recommend this procedure.

In 95.6% of patients, the symptoms controlled with the UAE helped to avoid major surgery. No major complication occurred.

### Strengths and limitations

This study's main strengths are the solid data collection and the unusual application of UAE in postmenopausal women.

The main limitation of the study is the small sample size, as it includes only 23 patients and that all these cases were performed by the same, very experienced, interventional radiologist. Therefore, these results cannot be generalizable to other units, and other practitioners. A large multicenter study would be needed to produce generalizable results.

### Interpretation

Our results show that this group of patients can be significantly helped by fibroid embolization.

We have shown that embolization in a select group of patients with an experienced radiologist is effective in terms of fibroid shrinkage and symptomatic relief. If embolization in these patients fails or does not provide symptomatic relief, then surgery is not precluded.

## Conclusions

A major and understandable concern of gynecologist has been the danger of uterine sarcomas in postmenopausal patients. We know, however, that sarcoma change in fibroids is extremely rare.^10,12–15^ Furthermore, we have the added diagnostic tools of MRI scan and guided biopsy procedures to ensure we have ruled out malignancy before UAE.

Our local protocol in patients where there may be an increased risk of sarcoma, for example, postmenopausal patients, or rapidly growing masses is to perform an MRI scan 4 weeks after the embolization to confirm that all the fibroid material is infarcted, in which case a sarcoma would be very rare.

The technique of UAE has evolved together with the experience of radiologists carrying out the procedure. It is questionable how valid some re-interventions and complication rate of early trials are in which patients were embolized by inexperienced radiologists using now outdated techniques. The most recent re-interventional rates are quoted 7% (12 months postprocedure) and 24% (5 years post-UAE).^16^

In conclusion, we have presented a series of older and postmenopausal patients who have undergone UAE: a group normally and historically excluded from this procedure. We have shown that in this patient group, UAE is extremely effective and should be strongly considered among the other surgical and nonsurgical options.

## Abbreviations Used

MRI: magnetic resonance imaging
UAE: uterine artery embolization
USS: ultrasound
